# Hepatic Atypical Protein Kinase C: An Inherited Survival-Longevity Gene that Now Fuels Insulin-Resistant Syndromes of Obesity, the Metabolic Syndrome and Type 2 Diabetes Mellitus

**DOI:** 10.3390/jcm3030724

**Published:** 2014-07-07

**Authors:** Robert V. Farese, Mackenzie C. Lee, Mini P. Sajan

**Affiliations:** 1 Research Service-108, James. A. Haley Veterans Medical Center, 13000 Bruce B. Downs Blvd., Tampa, FL 33612, USA; 2 Medical and Research Services, James A. Haley Veterans Medical Center, Tampa, FL 33612, USA; E-Mails: mackenzielee@gmail.com (M.C.L.); msajan@health.usf.edu (M.P.S.); 3 The Division of Endocrinology and Metabolism, Department of Internal Medicine, University of South Florida College of Medicine, Tampa, FL 33612, USA

**Keywords:** insulin resistance, obesity, type 2 diabetes mellitus

## Abstract

This review focuses on how insulin signals to metabolic processes in health, why this signaling is frequently deranged in Western/Westernized societies, how these derangements lead to, or abet development of, insulin-resistant states of obesity, the metabolic syndrome and type 2 diabetes mellitus, and what our options are for restoring insulin signaling, and glucose/lipid homeostasis. A central theme in this review is that excessive hepatic activity of an archetypal protein kinase enzyme, “atypical” protein kinase C (aPKC), plays a critically important role in the development of impaired glucose metabolism, systemic insulin resistance, and excessive hepatic production of glucose, lipids and proinflammatory factors that underlie clinical problems of glucose intolerance, obesity, hepatosteatosis, hyperlipidemia, and, ultimately, type 2 diabetes. The review suggests that normally inherited genes, in particular, the aPKC isoforms, that were important for survival and longevity in times of food scarcity are now liabilities in times of over-nutrition. Fortunately, new knowledge of insulin signaling mechanisms and how an aberration of excessive hepatic aPKC activation is induced by over-nutrition puts us in a position to target this aberration by diet and/or by specific inhibitors of hepatic aPKC.

## 1. An Overview and Darwinian Perspective of the Problem

Insulin-resistant syndromes of obesity, the metabolic syndrome and type 2 diabetes mellitus have reached pandemic levels in Western/Westernized societies. Although there have been efforts to find a genetic defect(s) that underlies this pandemic, given the breadth of the problem, it seems likely that humans, collectively, have inherited a set of genes that have been critical for survival, but nevertheless predispose us to develop insulin resistance, a key factor in the development of the aforesaid syndromes. Here, we propose that this predisposing gene set was critically needed for survival when we and our forbearers existed as hunter-gatherers, but now, in times of food surfeit, these genes are a root cause, or have abetted development, of the aforesaid insulin-resistant syndromes. We further propose that the predisposing nutrition-related survival genes include, perhaps most importantly, three largely homologous isoforms of “atypical” protein kinase C (aPKC), PKC-ι which is found only in primates, its mouse counterpart, PKC-λ, and the more ubiquitous isoform, PKC-ζ. These archetypal protein kinases phosphorylate many proteins that control diverse biological functions, ranging from cell growth and differentiation to inflammation and various metabolic and nutritional processes. Indeed, total knockout of PKC-λ in the mouse, and presumably PKC-ι in humans, is embryonically lethal.

As to the role the aPKCs in metabolism and nutrition, in the liver, during feeding, insulin activates both PKC-ι/λ and PKC-ζ, which, along with another insulin-activated protein kinase, Akt, increases hepatic lipid production from simple dietary precursors. The lipids that are produced in liver are released into the circulation for subsequent storage in adipose tissues. This process is critically needed for survival when food is scarce and feeding is sporadic. However, as we will discuss, this process becomes detrimental if over-utilized by over-nutrition.

With respect to glucose metabolism, insulin diminishes hepatic gluconeogenesis primarily by activating hepatic Akt, and this mechanism is essential for preventing the development of hyperglycemia. However, in marked contrast, hepatic aPKC, which is also activated by insulin, actually restrains the effects of hepatic Akt on gluconeogenesis. This implies that hepatic aPKC increases gluconeogenesis, which function is essential for maintaining brain and muscle function in fasting conditions. On the other hand, the activity of hepatic aPKC needs to be limited, *i.e.*, cannot be allowed to become excessive when nutrition is over-abundant, if we are to avoid excessive inhibition of Akt and subsequent development of glucose intolerance and diabetes mellitus. Unfortunately, hepatic aPKC is not only activated by insulin, but also by lipids derived directly from dietary carbohydrate and lipid precursors; moreover, as we will discuss, this activation of hepatic aPKC is clearly excessive with over-nutrition. Additionally, hepatic aPKC is further activated excessively by insulin itself as hyperinsulinemia develops in response to (a) excessive nutrition-dependent increases in insulin secretion from pancreatic islets; (b) inordinate increases in hepatic glucose production owing to mechanisms described herein; and/or (c) impairments in extrahepatic glucose disposal, particularly in muscle.

To add further insult to the above-described injuries in insulin-resistant hyperinsulinemic states, the human PKC-ι gene, unlike PKC-λ and PKC-ζ genes in subprimates, is activated by insulin-induced [1] and probably by lipid-induced increases in enzyme activity of aPKC. Accordingly, in insulin-resistant hyperinsulinemic conditions in humans, there is operation of a positive feedback loop in which hepatic aPKC activation provokes increases in transcription of the hepatic PKC-ι gene and subsequent increases in protein levels of hepatic PKC-ι, which, in turn, increase hepatic aPKC activity, and so on, to produce an auto-stimulatory vicious cycle.

Unfortunately, the increased activity of hepatic aPKC leads to excessive increases in transcription of a large array of lipogenic enzymes, and activation of factors that increase production of multiple proinflammatory cytokines. These liver-derived lipids and proinflammatory cytokines can enter the circulation and are most likely major contributors to the subsequent impairment of insulin signaling to both Akt and aPKC in muscle, which in turn leads to diminished glucose transport, subsequent decreases in whole-body glucose disposal, and a further impairment in glucose tolerance. Our experience in mouse obesity models is that treatment with agents that selectively target and improve, *i.e.*, normalize, hepatic aPKC leads to restoration of normal insulin signaling and action in muscle, even while high fat feeding continues.

In addition to promoting insulin resistance, excessive production of aPKC-dependent hepatic lipids leads to hepatosteatosis, and the excessive release of hepatic lipids leads to abdominal obesity, hypertriglyceridemia and hypercholesterolemia. In short, a full-blown metabolic syndrome ensues, and this sets the stage for development of type 2 diabetes mellitus.

To recapitulate, and, as will be discussed in greater detail below, with diet-induced excessive activation of hepatic aPKC, there is at first (presumably reflecting an early phase of obesity), an impairment in the ability of Akt to regulate (phosphorylate) downstream factors that diminish gluconeogenesis, and subsequent development of glucose intolerance, insulin resistance, hyperinsulinemia and increases in hepatic production of lipids and proinflammatory factors. As obesity progresses, the activation of Akt is itself impaired, causing further increases in hepatic glucose production and glucose intolerance. Later, when pancreatic insulin secretion is less able or can no longer compensate for impairments in glucose homeostasis, Akt activation diminishes even further and type 2 diabetes worsens.

## 2. Current Approaches for Controlling Insulin-Resistant Syndromes

Insulin-resistant syndromes of obesity, the metabolic syndrome and type 2 diabetes mellitus (T2DM) are estimated to be present in approximately 25% of adolescents and 50% of adults over the age of 50 in the United States, and their prevalence is on the rise as other populations are Westernized. Moreover, these syndromes, with attendant increases in blood pressure and serum levels of triglycerides, cholesterol, and proinflammatory cytokines, are responsible for much of the cardiovascular disease seen in Western/Westernized populations.

Unfortunately, to date, preventive measures and treatments for this pandemic have had limited success. Although diet and exercise programs, if properly executed, particularly during early stages, are effective, safe and economical, this approach is frequently not fruitful. Weight loss induced by bariatric surgery is frequently effective, at least in the short run, but this approach requires an expensive operative procedure and is not always long-lived.

With respect to pharmaceutical approaches, many anti-obesity agents have undesirable properties, their usefulness for weight control is frequently limited, and they generally have little impact on carbohydrate and lipid metabolism. Thiazolidinediones (TZDs) are true insulin-sensitizers and can improve hyperglycemia, but these agents tend to increase adiposity, have minimal effects on hyperlipidemia, cause fluid retention, and may increase cardiovascular risk. As a result, TZD usage has waned.

As for insulin secretagogues, sulfonylureas improve hyperglycemia, but can cause serious hypoglycemia, have little effect on hyperlipidemia and lipid abnormalities, and may increase cardiovascular risk. Incretins, such as glucagon-like peptide-1 (GLP-1) analogues and GLP-1/DPP4-peptidase inhibitors, can increase meal-related insulin secretion without increasing risk for hypoglycemia, and are useful for diminishing hyperglycemia. GLP-1 analogues may additionally improve obesity and hyperlipidemia. On the other hand, increases in effective circulating insulin levels in an insulin-resistant state, as discussed below, may have undesirable effects on lipids and proinflammatory cytokines, and theoretically may promote cancer progression.

Presently, the most frequently used agent for treating T2DM is metformin. This agent diminishes hepatic gluconeogenesis and thereby improves hyperglycemia. Metformin also improves insulin signaling in muscle, and improves glucose disposal therein. The mechanism whereby, metformin achieves blood glucose-lowering effects in liver and muscle, is only partly understood. Most salutary effects of metformin are attributed to activation of 5′-AMP-dependent protein kinase (AMPK) [2,3]. Although AMPK activation in some tissues is elicited solely by activation of liver kinase B1 (LKB1), in human hepatocytes, metformin uncouples oxidative phosphorylation, and subsequent increases in AMP (generated at the expense of ATP) appear to account for increases in AMPK activity [4]. AMPK activation by metformin and a substance commonly called AICAR improves insulin effects on expression of gluconeogenic enzymes, phosphoenolpyruvate carboxykinase (PEPCK), and glucose-6-phosphatase (G6Pase), in hepatocytes of type 2 diabetic humans [5] by a poorly understood mechanism. However, metformin and AICAR, apparently via AMPK activation, secondarily activate aPKC in human hepatocytes, and this unfortunately increases expression of lipogenic enzymes therein [5], presumably limiting improvements in lipids that would otherwise be present with simple AMPK activation. As discussed below, the activation of hepatic aPKC by metformin may also limit improvements in gluconeogenesis that would otherwise occur in the absence of hepatic aPKC activation. In short, co-activation of hepatic aPKC limits effectiveness of metformin; nevertheless, metformin is perhaps the best, and most frequently used, insulin-sensitizer that we presently have in our armamentarium.

Clearly, there is an urgent need to develop newer approaches for treating obesity, the metabolic syndrome and T2DM. Since insulin resistance owing to impaired glucose homeostasis is at the core of these problems, and, since aberrations in insulin signaling underlie these impairments in glucose homeostasis, there is a critical need to develop therapeutic options that directly deal with these aberrations in signaling. Fortunately, we have developed new insights into insulin signaling mechanisms that exist in health and disease, and this has allowed us to: (a) define what we believe is a key over-active pathogenetic signaling factor for development of insulin resistance; and (b) develop agents that target this factor and thereby improve glucose and lipid metabolism. As alluded to above, this potential therapeutic target that we have identified is itself activated by insulin, but, unfortunately, is also activated by certain lipids that are increased by dietary excesses of either carbohydrates or lipids. This target is aPKC, which we have found to be excessively activated in the liver in all insulin-resistant models that we have examined (see review data [6]). Before focusing on this target as a pathogenetic factor, and, thus, as a potential therapeutic option, we will review our understanding of how insulin signals to metabolic processes.

## 3. General Aspects of Insulin Signaling to aPKC and Akt

Insulin binds to external cell surface receptors that tyrosine-phosphorylate and thus activate two main intracellular substrates referred to as insulin receptor substrates (IRSs), IRS-1 and IRS-2, which attach to the inner side of the plasma membrane and activate an enzyme, a lipid/protein kinase, called phosphatidylinositol (PI) 3-kinase (PI3K) (see review [6]). PI3K in turn rapidly converts the plasma membrane phospholipid, PI-4,5-(PO_4_)_2_, to a more highly charged phospholipid, PI-3,4,5-(PO_4_)_3_ (PIP_3_). By virtue of its acidic D3-PO_4_ group, PIP_3_ binds directly to basic amino acids in several factors, including, Akt, aPKCs and phosphoinositide-dependent kinase-1 (PDK1). This co-localization facilitates PDK1-dependent phosphorylation of activation loop sites of both aPKC and Akt, e.g., threonine-308 in Akt and threonine-403 in PKC-λ. These initial phosphorylations then enable secondary phosphorylations at downstream sites, which, for aPKCs, occurs at the auto(trans)phosphorylation site, e.g., threonine-555 for PKC-ι, and, for Akt, occurs on serine-473, which is phosphorylated by a “PDK2”, now identified as mammalian target of rapamycin-C2 (mTORC2). Activation of aPKC additionally involves, perhaps most importantly, binding of PIP_3_ to basic arginine residues in an auto-inhibitory pseudosubstrate sequence in the regulatory domain of all aPKCs, and this causes dissociation of the pseudosubstrate sequence from the substrate-binding site in the catalytic domain [7]. This dissociation causes molecular unfolding and allows substrates to gain access to the active catalytic/kinase sites of the aPKCs. In effect, PIP_3_ is the main “second messenger” for insulin and mediates most of its metabolic actions.

## 4. Insulin Signaling to aPKC and Akt in Muscle

In muscle, both aPKC and Akt are required for insulin stimulation of glucose transport (see review [6]), and both kinases are activated by insulin through the activation of IRS-1/PI3K (the function of IRS-2/PI3K in muscle is unknown). Thus, as muscle IRS-1/PI3K is generally downregulated in obesity and T2DM, the activation of both Akt and aPKC in muscle is frequently diminished in these disorders. As to why muscle IRS-1/PI3K is downregulated in obesity will be discussed below in greater detail, but we recently found in early phases of experimental obesity induced by high fat feeding, that an abnormality in hepatic glucose production appears to be more primary than, and seems to account for, impairments in insulin signaling in muscle.

On the other hand, it is also clear that a primary defect in insulin action specifically in muscle, as has been produced experimentally by muscle-specific knockout of either the insulin receptor [8] or PKC-λ [9] can cause systemic insulin resistance, hyperinsulinemia and secondary increases in hepatic aPKC activity, that in turn lead to increases in lipogenic and gluconeogenic enzymes, and, thus, initially, to development of metabolic syndrome features of abdominal obesity, hepatosteatosis and hyperlipidemia, and, later, to development of overt T2DM [10]. In this regard, moreover, it appears that first-degree relatives of type 2 diabetic humans have an abnormality (? inborn) in insulin signaling and action in muscle that: (a) is unrelated to obesity; (b) does not involve signaling to and through Akt to a major substrate, AS160 [11]; and (c) may set the stage for development of T2DM, perhaps with another insult, such as caloric excess. However, whether muscle aPKC activation is disturbed in these muscles, or whether a defect unrelated to either aPKC or Akt is involved in these muscles of first-degree relatives of type 2 diabetic humans is still unsettled. In any case, given the fact that simple over-nutrition can trigger experimental obesity-diabetes, and, given the apparent importance of over-nutrition as a causative agent in the present pandemic of human obesity and T2DM, it seems likely that the over-nutrition mechanisms postulated herein for development of obesity and T2DM play a pre-eminent role in fueling the present obesity-diabetes pandemic.

## 5. Insulin Signaling to aPKC and Akt in Liver

In liver, although Akt is activated primarily by IRS-1/PI3K and to a lesser, but significant, extent by IRS-2/PI3K, aPKCs, in contrast, are activated exclusively by IRS-2/PI3K during insulin action [6]. Accordingly, and importantly, in livers of type 2 diabetic rodents [12] and in isolated hepatocytes of type 2 diabetic humans [1], whereas IRS-1 levels and IRS-1/PI3K activation are generally diminished, IRS-2 levels and IRS-2/PI3K activation are fully or better maintained, or, in many cases, excessive. Thus, insulin signaling in liver bifurcates in insulin-resistant states, and this bifurcation at least partly explains how insulin activation of aPKC is conserved or excessive at the same time that insulin activation of hepatic Akt is impaired in diabetic liver [1,6,12]. In turn, these decreases in Akt activation can account for increases in hepatic gluconeogenesis in T2DM, despite the fact that there are “paradoxical” increases in hepatic lipogenic and proinflammatory pathways owing to continued or heightened activation of hepatic aPKC, and possibly other PKCs that are elevated in livers of type 2 diabetic humans. In addition to the decreases in hepatic Akt activity that are seen in overt T2DM, we recently found [13] that there are decreases in the ability of fully activated Akt to specifically regulate gluconeogenesis in experimental diet-induced obesity in mice, as discussed further below.

Insulin signaling downstream of Akt and aPKC in liver is particularly important for understanding how metabolic abnormalities arise in obesity and T2DM. To begin with, it should be recalled that insulin diminishes gluconeogenesis at least partly if not largely by decreasing expression of phosphoenolpyruvate carboxykinase (PEPCK) and glucose-6-phosphatase (G6Pase), which increase hepatic glucose production and subsequent release from the liver, respectively. Insulin decreases transcription of these genes by phosphorylating the forkhead box O1 factor (FoxO1) (which interestingly promotes or longevity or survival in lower organisms), thereby diminishing the import of FoxO1 into the nucleus, where FoxO1 would otherwise facilitate (among other things) glucagon-dependent increases in transcription of gluconeogenic enzymes, PEPCK and G6Pase [14,15]. This inhibitory action of insulin on hepatic FoxO1, PEPCK, and G6Pase appears to be mediated solely by Akt [16]. Moreover, it appears that the action of hepatic Akt on FoxO1 phosphorylation is actually opposed by hepatic aPKC [1,5,10,13,17]. Indeed, we recently studied two experimental murine obesity models, viz., diet-induced obesity wherein in high-fat-feeding increases hepatic ceramide levels and thereby causes excessive activation of hepatic aPKC [13], and hereditarily obese ob/ob mice [18], wherein, the congenital absence of leptin leads to (a) increases in appetite and food intake, and (b) decreases in energy expenditure. In both obesity models, we found that hepatic Akt activity is in fact increased, presumably by hyperinsulinemia owing to increased hepatic gluconeogenesis and resultant glucose intolerance, and hepatic aPKC activity is increased, apparently owing to both hyperinsulinemia and dietary lipid-induced increases in hepatic ceramides that directly activate aPKC [13,18]. Most interestingly, despite increased hepatic Akt activity in these obesity models, the effect of Akt on FoxO1 phosphorylation and therefore gluconeogenic enzyme expression is compromised, and this leads to increases in hepatic glucose production, secondary increases in pancreatic insulin secretion, further increases in hepatic aPKC activity, and activation of aPKC-dependent lipogenic and proinflammatory factors. Furthermore, insulin signaling to IRS-1/PI3K, Akt and aPKC in muscle is secondarily downregulated, most likely through release of inhibitory lipids and proinflammatory substances, e.g., nuclear factor kappa-B(NFκB)-dependent tumor necrosis factor-alpha (TNF-α) and interleukin-1beta (IL-1β) [1,10,12,13,17] from the liver; as a result, muscle glucose transport is diminished, and glucose intolerance worsens.

Remarkably, in the above-described obesity models, inhibitors of hepatic aPKC (used in doses that only partially reduce hepatic aPKC activity) fully or largely restore: (a) the impairment in hepatic Akt-dependent FoxO1 phosphorylation; (b) excessive hepatic expression of gluconeogenic enzymes, PEPCK and G6Pase; and (c) insulin signaling to both Akt and aPKC in muscle [13,18]. As a result of these biochemical improvements, there are improvements in clinical abnormalities, including, glucose intolerance, obesity, hepatosteatosis, and hyperlipidemia (note that reversibility is more complete in the high fat fed model, and continued resistance in ob/ob mice may reflect persistence of the impairment in energy expenditure caused by leptin deficiency). Thus, as all of these abnormalities in the high fat model can be fully or largely reversed by either of two low molecular-weight agents that selectively target hepatic aPKC, this speaks strongly to the importance and primacy of hepatic aPKC in causing the impairment in hepatic Akt-dependent FoxO1 phosphorylation. The fact that muscle signaling to aPKC and Akt improve after use of liver-specific aPKC inhibitors also suggests that muscle defects follow hepatic defects in both obesity models. However, as to therapeusis, it should be emphasized that, in both obesity models, caloric intake is increased and diet is a major initiating factor for abnormalities in hepatic aPKC. Accordingly, dietary treatments should be similarly efficacious.

In contrast to gluconeogenesis, which insulin controls (*i.e.*, diminishes) through Akt, rather than aPKC, insulin effects on hepatic lipogenesis are mediated by both Akt [19,20] and aPKC [1,5,10,12,16,17,21,22] through proteolytic activation and subsequent increases in expression of sterol receptor element binding protein-1c (SREBP-1c) itself and 44 lipogenic enzymes. Akt activates this pathway through phosphorylation of mTOR1 and S6 kinase [20], but how these factors operate distally is uncertain. It is also uncertain how aPKC activates the lipogenic pathway. In any case, activation of SREBP-1c by either or both aPKC and Akt most likely involves direct or indirect phosphorylation of SREBP-1c, subsequent proteolysis, nuclear import of the active proteolytic fragment of SREBP-1c, and *trans*-activation of an array of lipogenic genes. Thus, in the above-described obesity models wherein hepatic activities of both Akt and aPKC are increased, both kinases may contribute to increases in hepatic lipogenesis and subsequent development of abdominal obesity, hepatosteatosis and hyperlipidemia.

However, as discussed above, as obesity advances and T2DM develops in response to a reversible or irreversible downturn in insulin secretion, the activation of hepatic IRS-1/PI3K, and thus of hepatic Akt, diminishes. Accordingly, whereas this downturn in Akt activity would contribute to further decreases in FoxO1 phosphorylation and increases in hepatic gluconeogenesis, the progression of the diabetic state may diminish the contribution of Akt to hepatic lipogenic enzyme expression. Nevertheless, any remaining increases in hepatic lipogenesis may continue to be supported by elevations in activities of hepatic IRS-2/PI3K and aPKC, which we found to be well maintained, even when diabetes is produced by experimental destruction of insulin-producing pancreatic islet cells [12,22] Whether other factors, e.g., conventional or novel PKCs (cPKCs or nPKCs), that activate S6 kinase are needed to substitute for Akt in maintaining hepatic lipogenesis in later stages of T2DM is uncertain. In any case, we found in hepatocytes of type 2 diabetic humans, dependence of hepatic lipogenesis on aPKC continues, and inhibitors of hepatic aPKC correct or improve the excessive expression of hepatic lipogenic enzymes and proinflammatory factors [1].

## 6. Activation of aPKCs by Diet-Derived Lipids

As alluded to, ceramide, a complex lipid that can be synthesized *de novo* from fatty acyl-CoA and sphingosine, or produced by breakdown of sphingomyelin, directly activates aPKC [23]. Moreover, hepatic ceramide is increased in the mouse model of diet-induced obesity in which dietary fat is increased from supplying 10% to either 40% or 60% of calories, and increases in hepatic ceramide are accompanied by increases in hepatic aPKC activity [13,24,25,26].

Increased ceramide levels in muscle have also been imputed as a cause of diminished insulin signaling to Akt and impaired glucose transport in obesity and T2DM. However, it should be noted that aPKC activity is also markedly suppressed in these muscles, and this is opposite to the fact that ceramide activates aPKC [23]. Thus, the impairment of insulin activation of aPKC that is ubiquitously seen in muscles of obese or diabetic animals or humans [6] cannot be ascribed to increases in muscle ceramide. On the other hand, increases in muscle ceramide may contribute to impairments in muscle IRS-1/PI3K and Akt activation in obesity and diabetes.

## 7. Insulin Signaling to Other PKCs through the *de Novo* Pathway

Insulin also activates cPKCs and nPKCs, particularly in liver and adipose tissues, by activating glycerol-3-phosphate/acyltransferase, which generates phosphatidic acid (PA) from glucose-derived glycerol-phosphate and fatty acyl-CoA, *i.e.*, the “*de novo*” phospholipid synthesis pathway [27,28]. PA is then rapidly converted to diacylglycerol (DAG), which activates cPKCs and nPKCs. This effect of insulin on *de novo* PA synthesis is not dependent on PI3K in some tissues [28], and it is unclear if this pathway is conserved in human diabetic liver. PA can also be increased by phospholipase D action, which insulin reportedly activates in liver [29]. In addition, note that increases in hepatic PA may also increase hepatic aPKC activity, as PA, like PIP_3_, is an acidic phospholipid that directly activates aPKC [6], albeit at reduced efficiency as compared to PIP_3_. Further, as discussed above, DAG-activated cPKCs and nPKCs, which are activated in livers of type 2 diabetic humans [30], may participate in maintaining the lipogenic pathway [31], perhaps by activating S6 kinase, and the proinflammatory pathway [32], in diabetic liver.

## 8. Activation of cPKCs and nPKCs by Diet-Derived Carbohydrates and Lipids

As with insulin, the *de novo* synthesis of PA and DAG can also be increased by increases in glucose [33] or carbohydrates that are metabolized to glucose, and by increases in lipids that increase availability of fatty acids. Increases in DAG induced either by insulin, glucose and/or fatty acids may then activate cPKCs and nPKCs, which are known to phosphorylate and diminish activity of the insulin receptor, and perhaps other insulin signaling factors. The cPKCs and nPKCs also appear to contribute importantly to development of glucose-dependent microvascular abnormalities in diabetes mellitus.

## 9. Levels of aPKCs in Tissues of Type 2 Diabetic Humans

As alluded to, we [34] and others [35] have found that aPKC levels, in particular PKC-ι [1], are diminished by approximately 50% in muscles of humans who have type 2 diabetes mellitus. However, in striking contrast to muscle, levels of PKC-ι are increased in hepatocytes of humans who have type 2 diabetes [1]. This difference in levels of PKC-ι in muscle and liver most likely reflects that: (a) expression (*i.e.*, mRNA production) of PKC-ι, but not other aPKCs, is stimulated by insulin-induced increases in aPKC enzyme activity [1], *i.e.*, there is a forward-feed, positive feedback mechanism that is operative and excessive in hyperinsulinemic conditions; and (b) whereas IRS-1/PI3K and thus aPKC activities in muscle are diminished in type 2 diabetes, IRS-2/PI3K and aPKC activities are elevated in hepatocytes of type 2 diabetic humans [1]. Thus, the differential control of aPKC activity in muscle and liver by upstream activators seems to account for observed differences in levels of PKC-ι mRNA and PKC-ι protein in these tissues of type 2 diabetic humans.

It is important to note that: (a) alterations in aPKC levels in both muscle and liver are not seen in rodent models of diabetes, as PKC-λ and PKC-ζ are not subject to positive feedback regulation; (b) human muscle contains primarily PKC-ι and a small amount of PKC-ζ [1,6]; (c) human liver contains substantial amounts of both PKC-ι and PKC-ζ [1,6]; (d) deficiency of aPKC in muscle and excess of aPKC in liver that are seen in human T2DM are not seen in human obesity [36]; and (e) the deficiency of aPKC in muscle and the excess of aPKC in liver greatly compound metabolic problems of T2DM in that both aberrations increase blood glucose levels and therefore increase plasma insulin levels, hepatic aPKC activity and expression of hepatic lipogenic, gluconeogenic, and proinflammatory factors; in turn, hepatic abnormalities downregulates insulin signaling in muscle, and *vice versa*, and so on, in another vicious cycle.

## 10. Use of Inhibitors of Hepatic aPKC for Treatment of Obesity and T2DM

As stated above, the vicious cycle of excessive hepatic aPKC activation that exists in obesity and T2DM can be broken by selective inhibition of hepatic aPKC following either: (a) by dietary measures that diminish aPKC-stimulating effects of certain lipids and hyperinsulinemia; and (b) by treatment with liver-selective inhibitors of aPKC. To the latter end, in our initial studies of rodent models of obesity (viz., high fat fed mice and heterozygous muscle-specific PKC-λ knockout mice) [17] and T2DM (viz., Goto-Kakizaki rats and obese-diabetic ob/ob mice) [12], we used intravenous injection of an adenovirus expressing kinase-inactive aPKC, which rapidly (within 5 days) and selectively (because of adenoviral localization to liver) diminished hepatic aPKC activity by approximately 50%. With this partial decrease in hepatic aPKC activity, the heightened expression/activation of hepatic SREBP-1c and NFκB were markedly improved, and there were improvements of hepatosteatosis, hypertriglyceridemia, hyper-fattyacidemia, hypercholesterolemia and hyperinsulinemia. Additionally, insulin signaling in muscle, adipose tissue and liver improved, fasting levels of hepatic PEPCK/G6Pase diminished, and, with these alterations, glucose homeostasis markedly improved [12,17]. In addition, excessive expression of lipogenic and proinflammatory factors improved [12,17].

Subsequently, we used two low molecular weight chemical inhibitors of PKC-viz. “ICAPP”, 1*H*-imidazole-4-carboxamide,5-amino-1-2,3-dihydroxy-4-[(phosphono-oxy)methyl]cyclopentyl-1R-(1a,2b,3b,4a), which binds tightly to the substrate-binding site of PKC-ι/λ, and “ATM”, aurothiomalate, which binds to the PB1-binding site in the regulatory domains of all aPKCs, which is essential for activation and certain subsequent actions of aPKCs. We first used these inhibitors in isolated hepatocytes of type 2 diabetic humans [1] to verify that these inhibitors diminish overactive lipogenic, proinflammatory and gluconeogenic pathways. We then used them to treat heterozygous muscle-specific PKC-λ knockout mice that have abdominal obesity, a full-blown metabolic syndrome and T2DM [9,10]. Importantly, both inhibitors selectively inhibited hepatic aPKC and corrected the exaggerated increases in expression/activities of SREBP-1c, NFκB, lipogenic enzymes, fatty acid synthase (FAS) and acetyl-CoA Carboxylase (ACC), and proinflammatory cytokines, tumor necrosis factor-α (TNF-α) and Interleukin-1β (IL-1β), and gluconeogenic enzymes, PEPCK and G6Pase, both in isolated hepatocytes of type 2 diabetic humans [1] and livers of obese-type 2 diabetic heterozygous muscle-specific PKC-λ knockout mice [10]. Moreover, within seven days of treatment of the heterozygous muscle-specific PKC-λ knockout mice, there were dramatic reversals of abdominal obesity, hepatosteatosis, hypertriglyceridemia, fattyacidemia, hypercholesterolemia, hyperinsulinemia and hyperglycemia [10]. Furthermore, insulin signaling to Akt was improved in muscle, adipose and liver tissues, and insulin-stimulated aPKC activation was improved in muscle and adipose tissues [10].

It should be noted that ICAPP is a highly specific inhibitor of PKC-ι/λ, and has no effect on other PKCs, including, PKC-ζ, PKC-α, PKC-β, PKC-δ, PKC-ε, and PKC-θ, and does not alter AMPK or Akt activity or their activation [1]. Additionally, note that both ICAPP and ATM selectively target hepatic aPKC activity, and, when used *in vivo*, do not inhibit muscle or adipose tissue aPKC activity or their activation by insulin. In fact, other than hepatic aPKC, all other signaling effects of insulin on both aPKC and Akt are improved in muscle, liver and adipose tissues of heterozygous muscle-specific PKC-λ knockout mice following ICAPP or ATM treatment [10].

More recently, we used another low molecular weight agent, 2-acetyl-1,3-diketo-cyclopentane (ACPD), that inhibits both PKC-ι/λ and PKC-ζ with equal potency (I_50_, approximately 0.5 μM), but not cPKCs or nPKCs [5]. Like ICAPP and ATM, ACPD inhibits insulin-induced increases in aPKC activity and aPKC-dependent increases in lipogenic enzymes in human hepatocytes [5], and lipogenic and gluconeogenic enzymes and proinflammatory factors in livers of obese ob/ob mice [7], and high fat fed mice [13]. Also, like ICAPP and ATM, ACPD does not inhibit Akt and has no effect on activity or activation of AMPK [5,13]. Most interestingly, ACPD was used over a 10-week period in high fat fed mice [13] and ob/ob mice [18] with no obvious ill effects, and was very effective in improving hepatic FoxO1 phosphorylation and diminishing expression of gluconeogenic and lipogenic enzymes. In addition, and presumably secondary to hepatic alterations, insulin signaling to both Akt and aPKC in muscle improved, and clinical parameters, including, glucose tolerance, obesity, hepatosteatosis and hyperlipidemia, improved in both obesity models.

Finally, we have studied mice in which one PKC-λ was knocked out by standard homologous recombination methods, and these mice surprisingly had a global defect in insulin signaling to both Akt and aPKC in liver, muscle, adipose tissue, but, nevertheless, were not diabetic, had normal responsiveness to insulin in hyperinsulinemic-euglycemic clamp studies, and, moreover, were fully protected from developing glucose intolerance or insulin resistance, or lipid abnormalities, when challenged with a high fat diet [37]. Thus, partial deficiency of aPKC throughout the body conferred remarkable metabolic protection.

## 11. Conclusions

**Figure 1:**
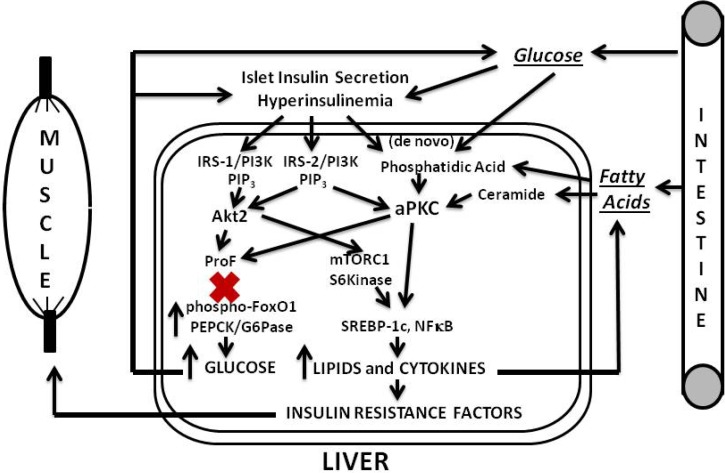
Phase 1 of insulin resistance (early diet-induced obesity).

**Figure 2:**
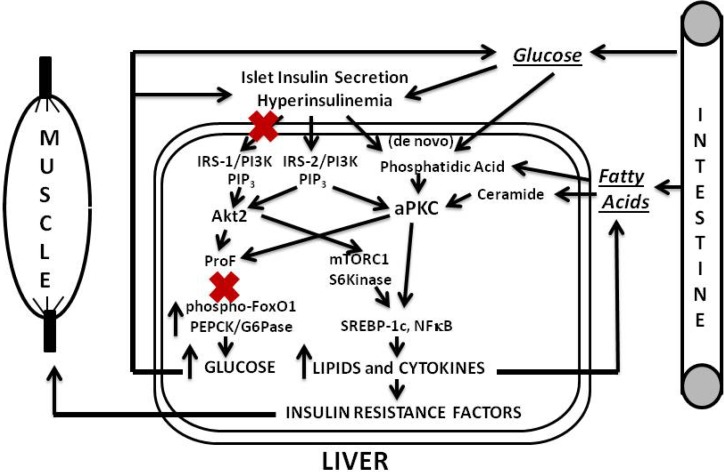
Phase 2 of insulin resistance (later diet-induced obesity and early T2DM).

**Figure 3:**
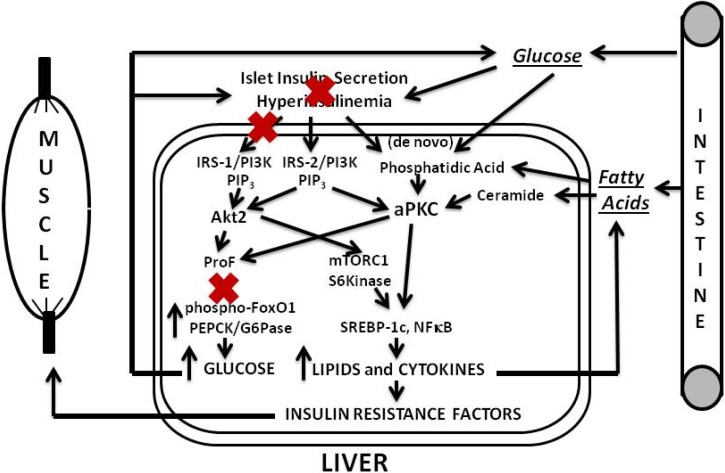
Phase 3 of insulin resistance (later T2DM).

Our findings and postulates are summarized in Figure 1, Figure 2 and Figure 3. In the first phase of insulin resistance, as seen in mouse models of diet-dependent obesity that are produced either by a moderate increase in dietary fat or carbohydrate intake, or by hyperphagia secondary to genetic (ob/ob) leptin deficiency, hepatic aPKC activity is inordinately increased, and, presumably because of co-localization of aPKC, Akt and FoxO1on the same ProF platform [13,38,39], and because of the ability of aPKC to bind, phosphorylate and inhibit Akt [40,41,42], the effects of Akt on FoxO1 phosphorylation are selectively diminished [13]. This selective impairment in FoxO1 phosphorylation leads to increased expression of gluconeogenic enzymes, glucose intolerance, hyperinsulinemia, further activation of hepatic aPKC and Akt, and further increases in expression of lipogenic enzymes and proinflammatory factors. Even in this early phase of insulin resistance, as well as in later phases, increases in hepatic production of lipid enzymes and proinflammatory factors appear to play important roles in causing decreases in insulin signaling to IRS-1/PI3K, aPKC and Akt in muscle, which in turn causes further worsening of glucose tolerance and insulin resistance, and thus further activation of hepatic aPKC and increases in production of hepatic lipid and proinflammatory factors.

In later phases of diet-induced obesity and early phases of type 2 diabetes, increases in hepatic aPKC activity are further heightened, and defects in activation of hepatic IRS-1/PI3K and Akt develop, further diminishing FoxO1 phosphorylation, and further increasing expression of gluconeogenic enzymes. Lipogenic enzyme expression and production of proinflammatory factors are still elevated by actions of aPKCs, residual Akt and perhaps other activators of S6 protein kinase.

In later phases of T2DM, pancreatic islet insulin secretion continues to fall, and this causes further decreases in hepatic IRS-1/PI3K and Akt, and further increases in hepatic gluconeogenesis. However, IRS-2/PI3K activation is more resistant to downregulation, and, along with increases in lipids that directly activate aPKCs (and, for that matter, DAG-dependent cPKCs and nPKCs), aPKC-dependent lipogenic and proinflammatory pathways may be better maintained. Of course, if insulin secretion is fully compromised, as in very late stages of T2DM, insulin activation of IRS-2/PI3K and IRS-2-dependent aPKC would also diminish. Nevertheless, IRS-2, aPKC, and other PKC activities may continue to be maintained by stimulatory lipids derived from high circulating levels of glucose, and fatty acids, and possibly by other factors.

To add further insult in hepatocytes of type 2 diabetic humans, hepatic PKC-ι expression and actual protein levels of PKC-ι are increased by insulin (and possibly by lipids), and a vicious cycle of excessive activation and production of aPKC is set up, further intensifying all metabolic problems. With increases in circulating lipids and proinflammatory cytokines, and with increases in blood pressure that ensues from insulin resistance and hyperinsulinemia, cardiovascular risk is increased.

Fortunately, the vicious cycle of aPKC hyper-expression and hyper-activity in human hepatocytes can be broken by low molecular weight inhibitors of aPKC. And fortunately, as we have seen in rodent models, breaking of this vicious cycle can largely correct obesity, metabolic syndrome abnormalities and T2DM. Whether the present or yet-to-be-developed low molecular weight inhibitors of aPKC will prove to be as effective in humans as they have been in rodents remains to be determined. In addition, there is a critical need to address safety issues during long-term treatment with aPKC inhibitors. At this point, we have reason to believe that this pharmacologic approach will be useful. However, also note that simple incubation of hepatocytes of type 2 diabetic humans in the absence of insulin (and/or other factors) can break this vicious cycle [1], and this suggests that dietary measures that are effective in reversing hyperinsulinemia should be similarly effective.
